# Epigenetic inactivation of the CpG demethylase TET1 as a DNA methylation feedback loop in human cancers

**DOI:** 10.1038/srep26591

**Published:** 2016-05-26

**Authors:** Lili Li, Chen Li, Haitao Mao, Zhenfang Du, Wai Yee Chan, Paul Murray, Bing Luo, Anthony TC Chan, Tony SK Mok, Francis KL Chan, Richard F Ambinder, Qian Tao

**Affiliations:** 1 Cancer Epigenetics Laboratory, Department of Clinical Oncology, State Key Laboratory of Oncology in South China, Sir YK Pao Center for Cancer, Li Ka Shing Institute of Health Sciences, The Chinese University of Hong Kong, Hong Kong; 2 School of Biomedical Sciences, The Chinese University of Hong Kong, Hong Kong; 3 School of Cancer Sciences, University of Birmingham, Birmingham, UK; 4 Department of Medical Microbiology, Qingdao University Medical College, Shandong, China; 5 Institute of Digestive Disease and State Key Laboratory of Digestive Diseases, Department of Medicine and Therapeutics, The Chinese University of Hong Kong, Hong Kong; 6 Johns Hopkins Singapore and Sydney Kimmel Comprehensive Cancer Center, Johns Hopkins School of Medicine, Baltimore, USA.

## Abstract

Promoter CpG methylation is a fundamental regulatory process of gene expression. TET proteins are active CpG demethylases converting 5-methylcytosine to 5-hydroxymethylcytosine, with loss of 5 hmC as an epigenetic hallmark of cancers, indicating critical roles of TET proteins in epigenetic tumorigenesis. Through analysis of tumor methylomes, we discovered *TET1* as a methylated target, and further confirmed its frequent downregulation/methylation in cell lines and primary tumors of multiple carcinomas and lymphomas, including nasopharyngeal, esophageal, gastric, colorectal, renal, breast and cervical carcinomas, as well as non-Hodgkin, Hodgkin and nasal natural killer/T-cell lymphomas, although all three *TET* family genes are ubiquitously expressed in normal tissues. Ectopic expression of TET1 catalytic domain suppressed colony formation and induced apoptosis of tumor cells of multiple tissue types, supporting its role as a broad *bona fide* tumor suppressor. Furthermore, TET1 catalytic domain possessed demethylase activity in cancer cells, being able to inhibit the CpG methylation of tumor suppressor gene (TSG) promoters and reactivate their expression, such as *SLIT2, ZNF382* and *HOXA9*. As only infrequent mutations of *TET1* have been reported, compared to *TET2*, epigenetic silencing therefore appears to be the dominant mechanism for *TET1* inactivation in cancers, which also forms a feedback loop of CpG methylation during tumorigenesis.

DNA methylation at the C5 position of cytosine (5-methylcytosine, 5-mC), known as the “fifth base”, is a key epigenetic modification at CpG dinucleotides, playing critical roles in normal development and disease pathogenesis including tumorigenesis^1^. Regional promoter CpG methylation together with genome-wide hypomethylation, as a fundamental epigenetic hallmark of cancers, lead to the silencing of tumor suppressor genes (TSG) and activation of oncogenes, contributing to cancer initiation and progression. Recently, various whole-genome sequencing studies of virtually all human cancers also demonstrate that the most commonly mutated genes are epigenetic modifiers including CpG methylation machinery components across diverse cancers^2^,^3^,^4^,^5^, highlighting the direct and crucial involvement of epigenetic programming dysregulation in tumorigenesis.

DNA methylation is a reversible process, through either passive or active demethylation. Passive demethylation has been well-documented owing to reduction in activities or absence of DNA methyltransferases (DNMTs) during DNA replication. The newly identified 5-hydroxymethylcytosine (5 hmC) in mammalian genomic DNA^6^, as an intermediate of active DNA demethylation, has been recognized as the “sixth base”, which provides us new insight into the regulation of CpG methylation dynamics via active demethylation. 5 hmC is readily expressed in human normal tissues and embryonic stem cells, but becomes greatly decreased in multiple cancer tissues^7^,^8^,^9^. 5 hmC modification is relatively stable, not just as a transient intermediate^10^, arising as a novel epigenetic hallmark of tumors^11^.

The ten-eleven translocation (TET) family of DNA hydroxylases, including TET1, TET2, and TET3, mediates the conversion of 5 mC to 5 hmC and final DNA demethylation through sequential oxidation reactions, thus as key executers for establishing 5 hmC pattern and maintaining a hypomethylated genome state^12^,^13^. *TET1* was firstly identified as a fusion partner of MLL in acute myeloid leukemia (AML)^6^. Inactive mutations or deletions of *TET2* with impaired catalytic activity were frequently detected in hematopoietic malignancies^14^, along with decreased 5 hmC levels^4^,^15^,^16^, while no somatic *TET1* or *TET3* mutation was found in myeloid and lymphoid tumors. The biological functions of TET family members or 5 hmC on the reprogramming and development of embryotic stem cells have been extensively studied^17^,^18^,^19^,^20^,^21^. Recent reports also demonstrate that *TET* gene expression are reduced in some solid tumors, associated with 5 hmC depletion and gene downregulation, thus playing critical functional roles in tumor initiation and metastasis^22^,^23^,^24^,^25^,^26^. Some mechanisms have been proposed to mediate TET disruption in cancers, including post-transcriptional regulation by miR-22^27^, post-translational modification by cellular proteolytic system^28^, and nuclear exclusion of TET proteins^29^,^30^. However, a systematic study of the expression and transcriptional regulation of TET members in most human cancers is still needed.

Here, we have studied the expression and transcriptional regulation of *TET* family genes in a large collection of human normal and tumor samples. We examined the epigenetic and genetic alterations of *TET1* through analyzing cancer methylomes previously established by us^31^ and also online genomics database of common tumors. We discovered frequent promoter methylation of *TET1* in a large set of tumor cell lines and primary tumors, and confirmed its tumor suppressive functions and demethylation activity in tumor cells.

## Results and Discussion

### Epigenomic identification of *TET1* as a methylated target in multiple cancers

**Figure 1:**
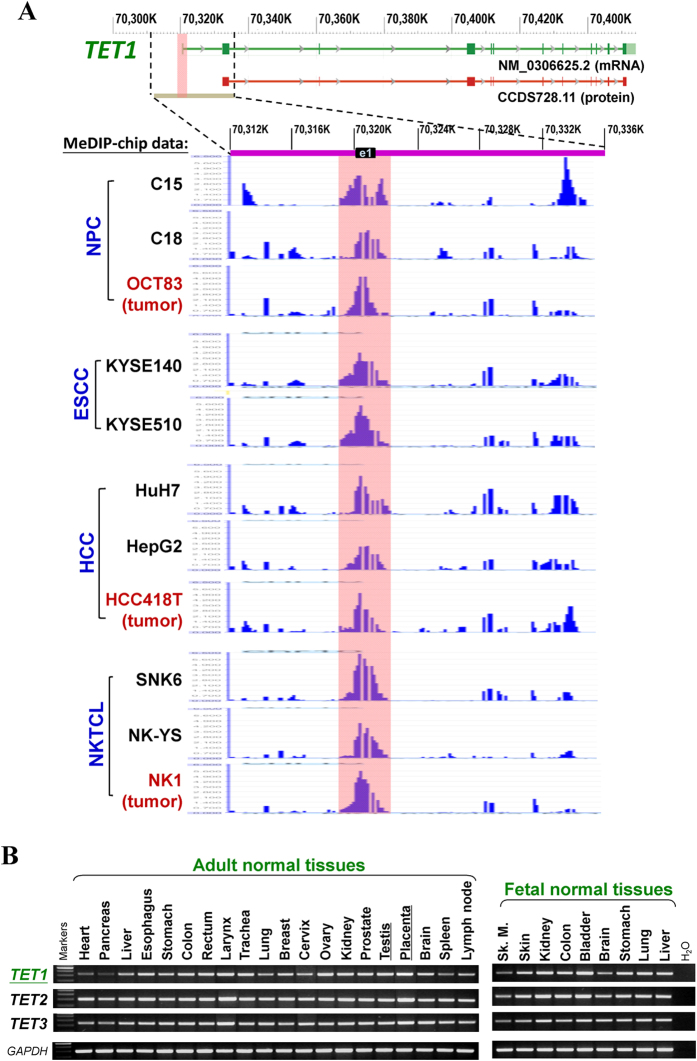
CpG methylome study identified *TET1* as a methylated target in multiple cancers. (**A**) Representative methylome data. *TET1* gene structure, promoter and exon 1 (NCBI database GRCh37.p13) are shown on the top panel. E1: exon 1. Positive methylation signal peaks identified by MeDIP-chip are shown in pink shadow for: NPC xenografts (C15, C18) and primary tumor (OCT83), ESCC cell lines (KYSE140, KYSE510), HCC cell lines (HuH7, HepG2) and primary tumor (HCC418T), NKTCL cell lines (SNK6, NK-YS) and primary tumor (NK1). (**B**) Expression of *TET* family genes (*TET1, −2, −3*) in human normal adult and fetal tissues by semi-quantitative RT-PCR, with *GAPDH* as a control. Sk. M., skeleton muscle.

**Figure 2:**
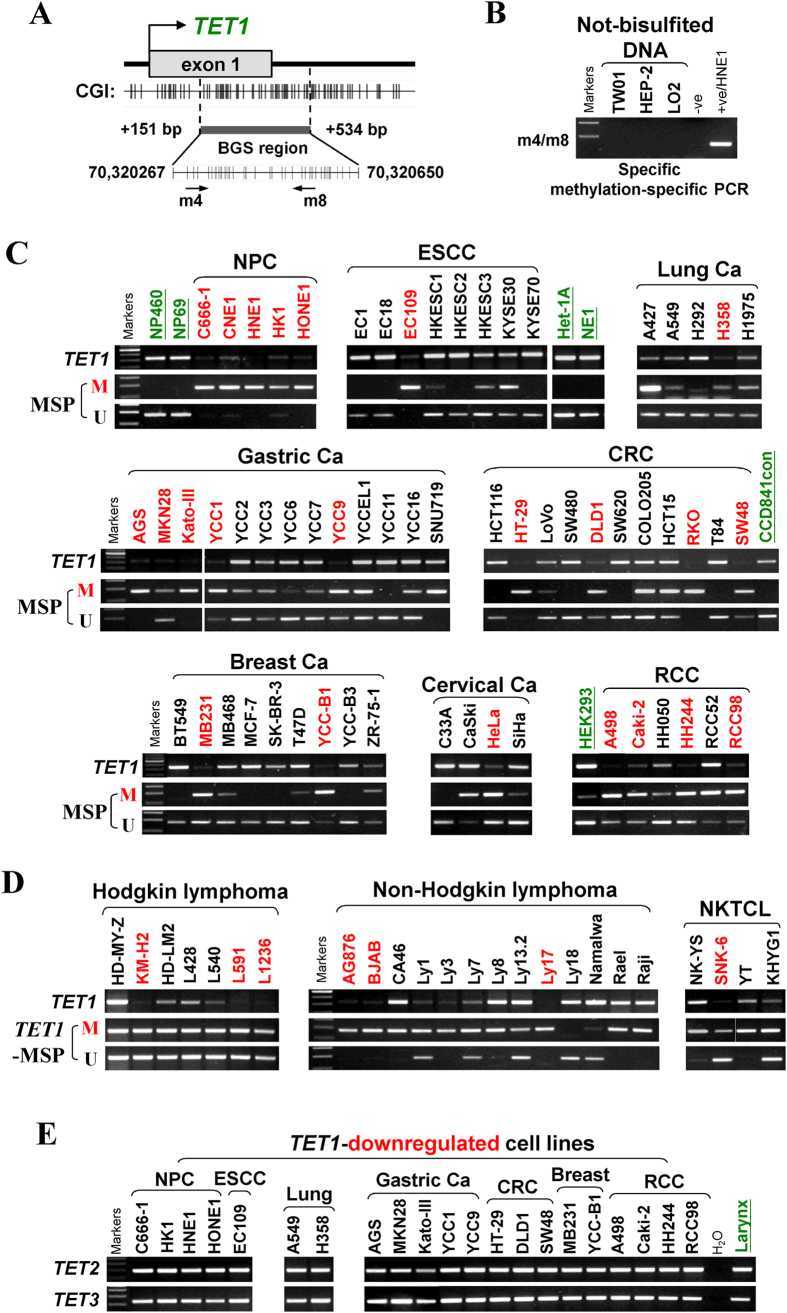
*TET1* is downregulated and methylated in multiple cancers. (**A**) Structure of the *TET1* promoter CpG island (CGI). CpG sites are shown as short vertical lines. MSP primer sites and BGS region analyzed are also indicated. (**B**) *TET1* methylation was not detected in not-bisulfited DNA samples, indicating that the MSP system is specific. m4/m8 represents specific MSP primer set of *TET1* methylation detection. (**C**,**D**) *TET1* was frequently silenced and methylated in multiple carcinoma and lymphoma cell lines, detected by semi-quantitative RT-PCR and MSP, but expressed and unmethylated in immortalized but non-transformed normal epithelial cell lines (with names green underlined). M, methylated; U, unmethylated. (**E**) Abundant expression of *TET2* and *TET3* in *TET1*-downregulated tumor cell lines. Ca, carcinoma; NPC, nasopharyngeal carcinoma; ESCC, esophageal squamous cell carcinoma; CRC, colorectal cancer; RCC, renal cancer; NKTCL, nasal NK/T-cell lymphoma.

During our analysis of whole-genome CpG methylation profiles (methylomes) of multiple tumor cell lines and primary tumors^31^, the promoter of one of the CpG demethylases, *TET1*, turned out to be a target in multiple methylomes (Fig. 1A). Bioinformatics analysis of the methylome data showed significant positive enrichment of CpG methylation (Cut off = 2) at the *TET1* promoter and exon 1 region in multiple tumors, including nasopharyngeal carcinoma (NPC) xenografts (C15, C18) and primary tumor (OCT83), esophageal squamous cell carcinoma (ESCC) cell lines (KYSE140, KYSE510), hepatocellular carcinoma (HCC) cell lines (HuH7, HepG2) and primary tumor (418T), as well as nasal NK/T-cell lymphoma (NKTCL) cell lines (SNK6, NK-YS) and primary tumor (NK1) (Fig. 1A). The *TET1* promoter and exon 1 region contain a typical CpG island (Fig. 2A), indicating that CpG methylation most likely regulates its expression in human cells.

We thus further examined the expression and methylation profiles of *TET1* in multiple cancers. Results showed that, although all three *TET* genes (*TET1, −2, −3*) were ubiquitously expressed in a series of human normal adult and fetal tissues (Fig. 1B), only *TET1* neither *TET2* nor *TET3*, was frequently downregulated or totally silenced in a variety of tumor cell lines including multiple carcinomas (nasopharyngeal, esophageal, lung, gastric, colon, breast, cervical, renal) and lymphomas (Hodgkin, non-Hodgkin and NKTCL), while *TET1* is readily expressed in all immortalized normal epithelial cell lines of different tissue origins (Fig. 2 and Suppl. Fig. S1A).

Methylation-specific PCR (MSP) primers for *TET1* was tested for not amplifying any not-bisulfited DNA, confirming the detection specificity of *TET1* methylation in our study (Fig. 2B). Then by MSP, we detected *TET1* promoter methylation in virtually all downregulated cell lines of nasopharyngeal, esophageal, lung, gastric, colon, breast, cervical and renal carcinomas, as well as Hodgkin (HL), non-Hodgkin (NHL) and NKTCL lymphomas, but not in immortalized normal epithelial cell lines (Fig. 2C,D; Table 1). Moreover, *TET1* downregulation and methylation were infrequently detected in hepatocellular (HCC) and prostate cancer cell lines but not in the bladder and melanoma cell lines examined (Suppl. Fig. S1B).

**Figure 3:**
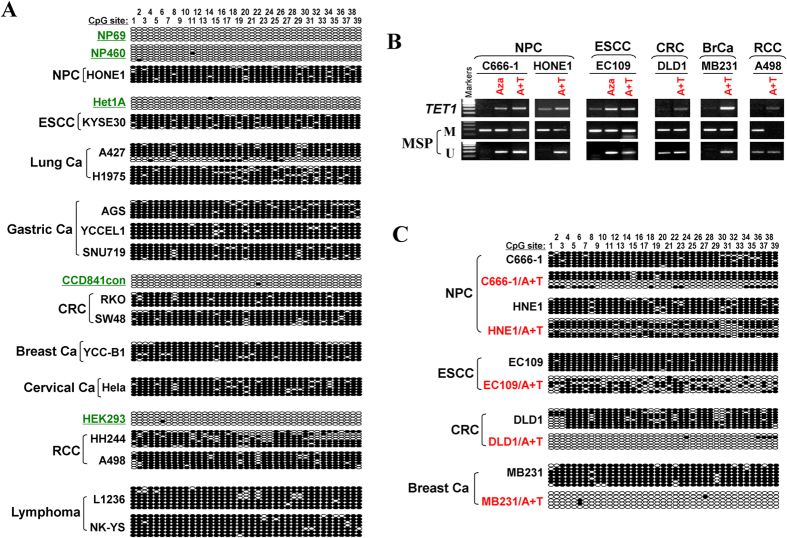
Demethylation treatment could reactivate *TET1* expression in silenced tumor cell lines. (**A**) Detection of *TET1* methylation in multiple tumor cell lines and normal cell lines by BGS. (**B**) Treatment with Aza or combined with TSA (A + T) demethylated *TET1* promoter in silenced cell lines of multiple tissue types. Expression and methylation changes were detected by semi-quantitative RT-PCR and MSP. (**C**) BGS analysis of *TET1* promoter in cell lines with or without treatment. NPC, nasopharyngeal carcinoma; ESCC, esophageal squamous cell carcinoma; CRC, colorectal cancer; BrCa, breast cancer; RCC, renal cancer; Ca, carcinoma.

We further studied the detailed methylation profile of *TET1* promoter by bisulfite genomic sequencing (BGS). A 384-bp region (+151-bp to +534-bp) spanning *TET1* promoter and exon 1, containing 39 CpG sites was analyzed (Fig. 2A). BGS results showed heavily methylated alleles in representative cell lines, including NPC, ESCC, lung, gastric, colon, breast, cervical and renal carcinomas, as well as lymphomas, while barely present in immortalized normal cell lines of nasopharyngeal (NP69, NP460), esophageal (Het-1A), colon (CCD841con) and kidney (HEK293) epithelial cells, consistent with the MSP data (Fig. 3A). Thus, *TET1* silencing by promoter CpG methylation is a common event in multiple tumors.

We further investigated whether *TET1* promoter methylation directly mediates its repression. DNA methyltransferase inhibitor 5-aza-dC (Aza) was used or in combination with histone deacetylase (HDAC) inhibitor to treat tumor cell lines of nasopharyngeal, esophageal, colon, breast and renal, all with methylated and downregulated *TET1.* After the treatment, restoration of *TET1* expression was observed, along with increased unmethylated promoter alleles as detected by MSP (Fig. 3B). Demethylation of the *TET1* promoter was confirmed by BGS analysis, which shows dramatically demethylated CpG sites (Fig. 3C), indicating that CpG methylation directly mediates *TET1* silencing in tumor cells.

In this study, we demonstrated that epigenetic silencing is a common regulatory mechanism for *TET1* inactivation at the transcriptional level in multiple human cancers. Additional alternative mechanisms regulating expression and activities of TET family members have been reported^32^. For examples, high mobility group AT-hook 2 (HMGA2), a chromatin remodeling factor, suppresses *TET1* expression by directly binding to its promoter or indirectly through other components in breast cancer cells^24^. Polycomb repressive complex 2 (PRC2) mediates *Tet1* downregulation through H3K27me3 histone mark deposition^33^. PARP activity increases *TET1* expression levels through maintaining a permissive chromatin state^34^. miR-22 suppresses *TET* expression levels in breast cancer cells through directly targeting the 3′-untranslated regions (UTRs) of *TET* mRNAs^27^. As direct substrates of calpains (calcium-activated cysteine proteases), TET proteins also undergo calpain-mediated degradation^28^. Nuclear exclusion of TET1 and TET2 is significantly correlated with loss of 5mC in glioma and colon cancer^29^,^30^. Thus, TET expression could be regulated at multiple levels of transcription, post-transcription or post-translation in different cell context, although *TET1* silencing through promoter CpG methylation appears to be more common and predominant in multiple tumors.

### Frequent silencing of *TET1* by promoter methylation in primary tumors

**Figure 4:**
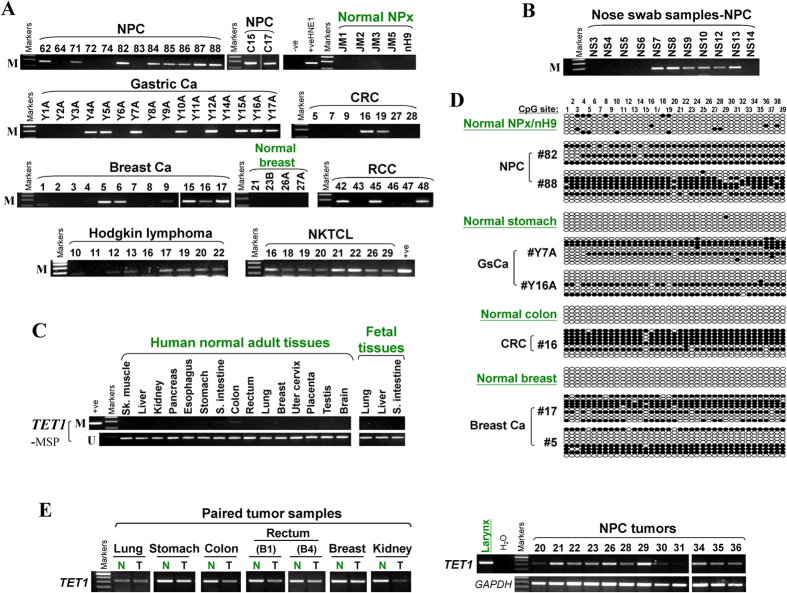
Frequent methylation of *TET1* in multiple primary tumors. *TET1* promoter methylation in (**A**) multiple primary tumors and (**B**) nose swab samples from NPC patients, detected by MSP. (**C**) *TET1* methylation is barely seen in normal tissues by MSP analysis. (**D**) Representative BGS analysis of *TET1* promoter methylation in primary tumors and normal tissues. Circles, CpG sites analyzed; row of circles, an individual promoter allele that was cloned, randomly selected and sequenced; filled circle, methylated CpG site; open circle, unmethylated site. (**E**) Levels of *TET1* mRNA expression in representative paired tumor (T)/normal (N) tissues, and primary tumor tissues (NPC), measured by semi-quantitative RT-PCR. Ca, carcinoma; NPC, nasopharyngeal carcinoma; CRC, colorectal cancer; RCC, renal cancer; GsCa, gastric cancer; Sk. muscle, skeleton muscle; S. intestine, small intestine.

As promoter CpG methylation in tumor cell lines might be derived from cell culture-induced secondary effect, we further examined *TET1* methylation and expression in primary tumor samples. We detected frequent *TET1* methylation in multiple tumors, including 55% (31/56) of NPC, 55% (30/55) of gastric, 27% (3/11) of colon, 42% (5/12) of hepatocellular, 36% (18/50) of breast and 28% (13/46) of renal tumor samples, as well as 78% of primary Hodgkin and 83% (10/12) of NKTCL lymphoma samples (Fig. 4A, Suppl. Fig. S2, Table 1), but infrequently in primary ESCC, lung, prostate tumors and other non-Hodgkin lymphomas (Suppl. Fig. S2, Table 1). *TET1* methylation could even be detected in 50% of 16 nose swab samples from suspected NPC patients (Fig. 4B). In contrast, *TET1* methylation was not detected in a panel of human normal adult and fetal tissues except for being barely seen in normal small intestine and colon (Fig. 4C). Further detailed BGS methylation analysis confirmed the presence of methylated promoter alleles in primary tumors but not normal tissues (Fig. 4D). *TET1* downregulation was also detected in paired primary tumors of several tissue types (lung, stomach, colon, rectum, breast and kidney) and primary NPC tumors (Fig. 4E). Furthermore, through online GENT and Oncomine database analysis, we found that *TET1* mRNA levels were significantly reduced in multiple solid tumors and leukemia, compared with their corresponding normal tissues (Suppl. Fig. S3). These results clearly demonstrate that *TET1* silencing by promoter CpG methylation is a common event for multiple tumors of epithelial and lymphoid origins.

Several studies have shown that *TET* genes are readily expressed in normal esophageal, gastric, colon, liver and breast tissues by PCR or immunohistochemistry^22^,^23^,^25^, but decreased in tumor cell lines and primary tumors to varied grades, with *TET1* as the most significantly downregulated member. A previous report through analyzing Cancer Genome Atlas TCGA database found that *TET1* is downregulated in primary tumors of colorectal, breast and lung since early stage, and associated with patient poor survival^23^. *TET1* is significantly decreased at mRNA and protein levels in gastric primary tumors compared to surgical margins and associated with tumor localization and TNM grades^35^. DNA methylation and bivalent histone marks at the CpG island 3′-shore mediate *TET1* silencing in gastric cancer^36^. Reduced TET1 expression or 5 hmC level in breast cancer tissues could be biomarkers for breast cancer progression^37^. *TET1* methylation in colorectal cancer tissues, not *TET2* and *TET3*^38^, has been found as an early event in CRC tumorigenesis, thus as a valuable biomarker for metastasis prediction^39^. Our results are consistent with these previous studies. *TET1* methylation appears to be tumor-specific and thus could serve as a potential epigenetic biomarker for cancer detection.

### Genetic alteration of *TET1* is uncommon in human cancers

**Figure 5:**
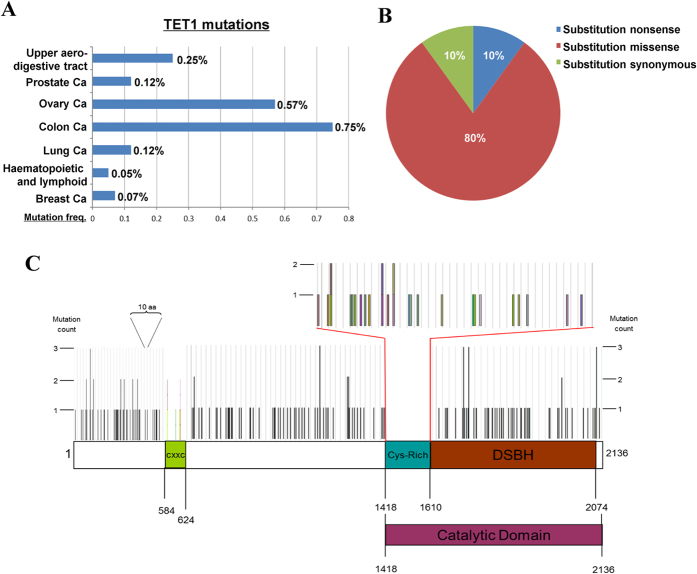
Gene mutation analysis of *TET1* in human cancers. Somatic mutations of *TET1* gene in human cancers were analyzed using the COSMIC database. (**A**) Frequencies and (**B**) distributions of *TET1* mutations. (**C**) Diagram displaying complete *TET1* mutation spectrum identified and their distribution in the coding region of TET1.

As alterations of cancer gene are through either genetic or epigenetic mechanisms, we further investigated possible genetic alterations of *TET1* in cancers. Somatically acquired mutations of *TET1* in human cancers were analyzed using the COSMIC database. Only <1% of tumor cases (most cases with ≤0.25%) had detectable *TET1* mutations (Fig. 5A), consisting of 80% of missense mutations, 10% of nonsense and 10% of synonymous mutations (Fig. 5B), with most of the mutations located in coding regions (Fig. 5C). We also detected hemizygous deletion of *TET1* in some tumor cell lines with *TET1* silencing and methylation, but not in *TET1*-expresssing cells (Suppl. Fig. S4A,B). Consistently, *TET1* gene deletion was also observed in solid tumors by analyzing DNA copy number alterations using the Oncomine database (Suppl. Fig. S4C). These results demonstrate that *TET1* mutation is uncommon in human cancers, although *TET1* deletion is indeed present in some tumor samples.

### TET1 functions as a tumor suppressor which requires its catalytic activity

**Figure 6:**
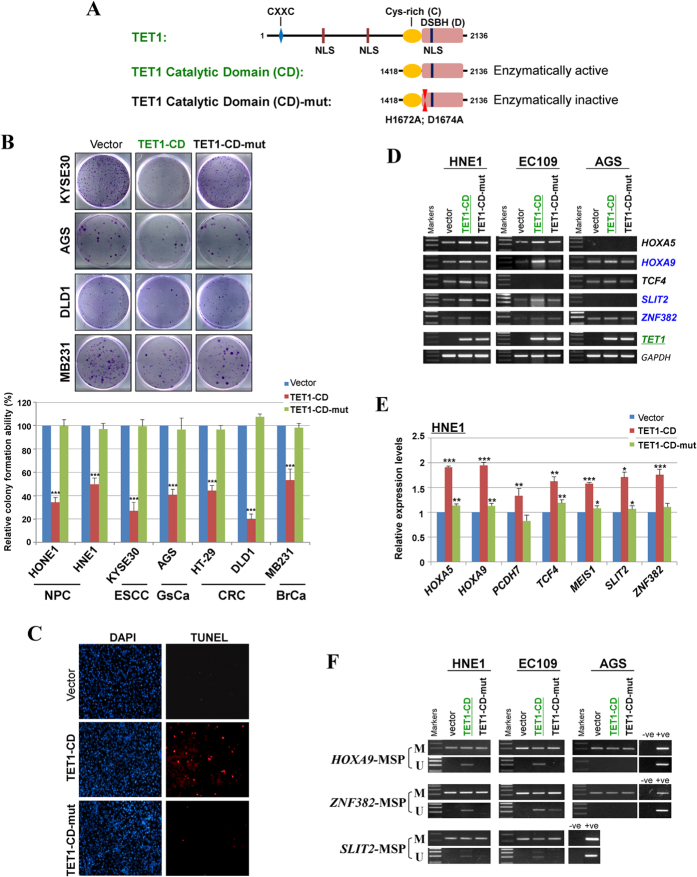
TET1 functions as a tumor suppressor in multiple tumor cells. (**A**) Structure and functional domains of the human TET1 protein, containing a C-terminal CD domain including the Cys-rich and DSBH regions, and a CXXC domain. The positions of three nuclear localization sequences (NLS) are shown. TET1 catalytic domain (TET1-CD) containing the Cys-rich and DSBH regions and TET1 mutant (TET1-CD-mut) with two amino acid substitutions (H1672A; D1674A) in the catalytic domain are also shown. (**B**) Ectopic expression of TET1-CD inhibited tumor cell growth of multiple tissue types. Representative colony formation assays of TET1-CD- and TET1-CD-mut-expressing tumor cells of nasopharyngeal, esophageal, gastric, colon, and breast cancers are shown. Quantitative analyses of colony numbers are shown as values of mean ± S.D. (lower panel), ***p < 0.001. NPC, nasopharyngeal carcinoma; ESCC, esophageal squamous cell carcinoma; GsCa, gastric cancer; CRC, colorectal cancer; BrCa, breast cancer. (**C**) Ectopic expression of TET1-CD induced tumor cell apoptosis. TET1-CD, TET1-CD-mut, and vector-expressing NPC tumor cells (HONE1) were analyzed by TUNEL assays. (**D**) TET1-CD upregulated multiple TSGs expression in tumor cells, as examined by semi-quantitative RT-PCR. (**E**) TET1-CD upregulated multiple TSGs expression as measured by qRT-PCR in NPC (HNE1) cells. Fold changes of TSGs expression in TET1-CD and TET1-CD-mut-transcfected cells were calculated by normalizing towards vector-expressing cells (set 1.0). *GAPDH* was used as an internal control. Data are shown as mean ± SD of three independent experiments. *p < 0.05; **p < 0.01; ***p < 0.001. (**F**) Detection of promoters methylation of *HOXA9, SLIT2* and *ZNF382* genes by MSP in TET1-CD and TET1-CD-mut-expressing tumor cells.

The TET1 catalytic domain (CD) (containing the Cys-rich and DSBH regions) remains intact hydroxylase activity in embryonic development and reprogramming^6^,^13^, displaying ability to induce 5 hmC formation, demethylation and gene transcription in differentiated cells^33^. We test whether the catalytic activity of TET1 was required for its possible tumor suppression functions, using TET1-CD and its enzymatic dead mutant (TET1-CD-mut) (Fig. 6A). Ectopic expression of TET1-CD significantly suppressed tumor cell clonogenicity (to ~40–50% of control cells) in colony formation assays of NPC, ESCC, gastric, colon and breast tumor cells, while the TET1-CD-mut lost this ability (Fig. 6B). TUNEL assay showed significantly increased numbers of apoptotic cells in TET1-CD expressing-tumor cells, compared with vector or TET1-CD-mut controls (Fig. 6C). These results demonstrate that TET1 possesses *bona fide* tumor suppressive functions in tumor cells of multiple types.

Consistent with our results, several recent studies have shown similar tumor-suppressive functions of *TET1* in cancer cells. TET1 inhibits proliferation and invasion of colon^23^, breast^24^,^25^, renal^40^ and prostate^25^ cancer cells *in vivo* and *in vitro. TET1* deficiency promotes B-lineage differentiation, leading eventually to B-cell lymphoma^41^. TET1 suppression as a key event of the RAS programming is required for KRAS-induced cellular transformation^26^. Thus, loss of function of TET1 is a common event during multiple tumorigenesis of solid tumors or hematologic malignancies.

### TET1 induces TSG promoter demethylation in tumor cells

Several studies identified TET1 target genes in mouse ES cells and some tumor cells, using RNA- or ChIP- sequencing or hydroxymethylated DNA immunoprecipitation sequencing (hMeDIP-seq)^12^,^24^,^26^,^27^,^33^,^42^,^43^,^44^,^45^. A series of TET1-targeted genes including TSGs have been identified, such as TIMP^25^, HOXA9 and HOXA7^24^, and Wnt signaling antagonists DKK3 and DKK4^23^. To further explore the molecular mechanism of TET1 in tumor suppression, we examined some known and potential target TSGs to assess the demethylase activity of TET1 in tumor cells. Mild upregulation of *HOXA9, HOXA5, PCDH7, TCF4, MEIS1, SLIT2* and *ZNF382* at mRNA levels was observed in TET1-CD-expressing carcinoma cells by semi-quantitative RT-PCR (Fig. 6D) and qRT-PCR (Fig. 6E). Meanwhile, we also detected decreased methylated alleles of *HOXA9, SLIT2* and *ZNF382* promoters in TET1-CD-expressing tumor cells, but not in TET1-CD-mut-expressing cells, with increased unmethylated promoter alleles observed concurrently, suggesting that TET1 indeed functions as a CpG demethylase to demethylate and reactivate multiple TSGs in tumor cells (Fig. 6F). In addition to *HOXA9*, we also found that TSGs like *SLIT2, ZNF382, PCDH7, TCF4, MEIS1* and *HOXA5* as TET1 target genes which could be demethylated and reactivated by TET1 in tumor cells. Other mechanisms besides demethylase activity could also be involved in regulating target genes by TET1, such as recruiting PRC2^42^, PRDM14^43^, Sin3A co-repressor complex^44^ and MBD3/NURD complex^45^. Further studies on TET1-targeted gene regulation in human cancers would help us to understand more of its role in cancer development.

**Figure 7:**
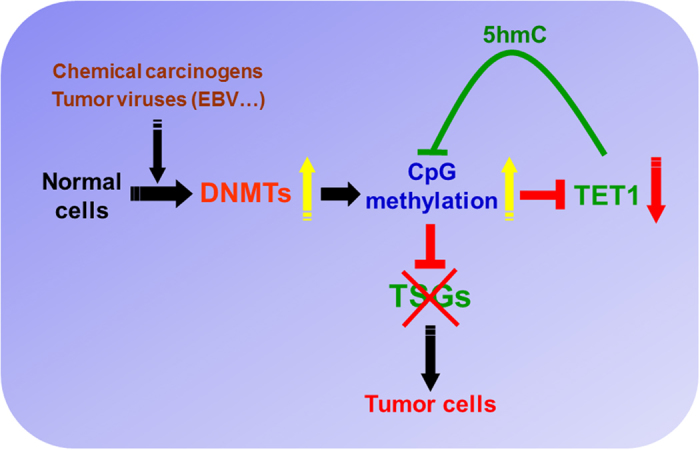
Proposed model of a DNA methylation feedback loop mediated by DNMTs/CpG methylation and TET1 during human tumorigenesis. When normal cells are exposed to carcinogens (chemical carcinogens, tumor viruses, etc), DNA methyltransferases (DNMTs) are induced, upregulated or overactivated, which further generates higher levels of DNA CpG methylation (5 mC). Elevated level of 5 mC on tumor suppressor gene (TSG) promoters lead to TSGs silencing and functional inactivation, ultimately to tumorigenesis. Ten-eleven-translocation (TET) proteins catalyze DNA CpG demethylation through converting 5 mC to 5-hydroxymethylcytosine (5 hmC), maintaining a delicate balance between CpG methylation and demethylation in normal cells. While in premalignant or tumor cells, CpG demethylation by TET would induce TSG promoter demethylation and functional restoration for further tumor suppression. Thus unlike normal cells where TET proteins are abundant, loss of *TET1* expression through promoter CpG methylation frequently occurs in tumor cells, which in turn, increases 5 mC levels and promotes TSG inactivation in tumor pathogenesis.

The discovery of TET enzymes, in addition to DNMTs, establishes a fundamental etiologic role of CpG methylation in human cancers. In response to environment carcinogens^46^,^47^,^48^ like chemical carcinogens and tumor viruses, DNMT activities and expression levels are induced and increased in cells, displaying stronger maintenance and *de novo* methylation capacity, leading to specific gene CpG island hypermethylation. The epigenetic alterations, especially promoter CpG methylation of TSGs, facilitate genome instability, disrupted cellular signaling and even further genetic mutations, thus are crucial to tumor initiation and progression^1^,^49^. Remarkably, promoter CpG methylation-mediated silencing of the CpG demethylase *TET1* in human cancers, which in turn, further leads to increased 5 mC levels in tumor cells, thus forming a DNA methylation feedback loop mediated by DNMT/CpG methylation and TET1 (Fig. 7).

In summary, our study comprehensively examined *TET1* expression and methylation status in multiple tumors, and demonstrated that promoter CpG methylation is a predominant mechanism for *TET1* inactivation in human cancers. The tumor-specific methylation of *TET1* could serve as a valuable, epigenetic non-invasive biomarker. TET1 as a tumor suppressor and CpG demethylase in tumor cells requires its intact catalytic domain, which provides new insight into the epigenetic master role of TET1 in tumor pathogenesis. Our findings enlighten us on the mechanistic elucidation of the importance of CpG methylation in human cancers.

## Material and Methods

### Cell lines and tissue samples

Human tumor cell lines of multiple tissue types were used^50^,^51^,^52^,^53^,^54^,^55^, including nasopharyngeal (NPC), esophageal squamous cell (ESCC), lung, gastric, colorectal (CRC), hepatocellular (HCC), breast, cervical, renal (RCC), bladder and prostate carcinomas, melanoma, as well as non-Hodgkin (NHL), Hodgkin (HL) and nasal natural killer (NK)/T-cell (NKTCL) lymphomas. Immortalized, non-transformed normal epithelial cell lines were used as “normal” controls. Cell lines were obtained from either American Type Culture Collection or collaborators. When needed, cell lines were treated with 10 μmol/L 5-aza-2′-deoxycytidine (Aza) (Sigma-aldrich, St Louis, MO) for 3 days, without or with further treatment with 100 nmol/l trichostatin A (TSA) (Cayman Chemical Co., Ann Arbor, MI) for additional ~16 h as previously^50^,^53^. Normal adult and fetal tissue RNA and DNA samples were purchased commercially (Stratagene, La Jolla, Ca; Millipore-Chemicon, Billerica, Ma). DNA samples of primary carcinomas, nose swab from suspected NPC patients, as well as surgical margin normal tissues, have been described previously^31^,^51^,^52^.

### Establishment of tumor methylomes by MeDIP-chip

Methylated DNA immunoprecipitation (MeDIP) coupled with promoter microarray hybridization was performed as previously^31^. Briefly, immunoprecipitation of methylated DNA was performed using monoclonal antibody against 5-methylcytidine (33D3, Diagenode, Seraing, Belgium) labeled with magnetic beads. Total input and immunoprecipitated DNA were labeled with Cy3 or Cy5, respectively, and hybridized to NimbleGen™ HG18 Meth (385K CGI plus) promoter arrays or HG19 (2.1 M) Deluxe Promoter arrays (Array Star, Inc., MD). Normal epithelial cell lines and normal tissues were used as controls. Bioinformatics analysis of methylome data was performed as previously^31^.

### Semi-quantitative RT-PCR and quantitative real-time PCR (qRT-PCR)

Semi-quantitative RT-PCR and quantitative real-time PCR were performed as described before^50^,^53^, with *GAPDH* as a control for all the samples shown in our previous publications^31^,^51^,^52^. qRT-PCR was carried out according to the manufacturer’s protocol (HT7900 system; applied Biosystems), with SYBR Green master mix (applied Biosystems) used. Primers used are listed in Supplementary Table S1.

### Bisulfite treatment of DNA samples and promoter methylation analysis

CpG island (CGI) analysis for *TET1* promoter and exon 1 was performed using CpG island Searcher (http//ccnt.hsc.usc.edu/cpgislands2). Bisulfite modification of genomic DNA was carried out as described previously^56^,^57^. For MSP analysis, approximately 50 ng of bisulfited DNA for each sample was amplified with methylation- or unmethylation- specific primer set, according to our previous MSP protocol^58^. Bisulfite-treated DNA was also amplified using a set of BGS primers, then cloned into pCR4-TOPO vector (Invitrogen, Carlsbad, Ca), with 8–10 clones randomly picked and sequenced. MSP and BGS primers used are shown in Supplementary Table S1. Unmethylated gene alleles for these treated samples have been detected in our previous publications, which shows the good quality of these DNA samples^31^,^51^,^52^.

### Genetic deletion analysis for *TET1*

Homozygous deletion of *TET1* coding exons 2 and 4 was examined using multiplex genomic DNA PCR, as previously described^51^. Primer sequences are shown in Supplementary Table S1.

### Colony formation assay of tumor cells

Human TET1 catalytic domain (TET1-CD) cDNA and its catalytic domain mutant (TET1- CD-mut) clones (Addgene, Cambridge, MA) were used as templates to generate TET1 constructs with an N-terminal Flag tag, and subcloned into pcDNA3.1 vector (Invitrogen, Carlsbad, Ca). Cells were cultured overnight in a 12-well plate and transfected with empty vector or TET1-CD, TET1-CD-mut-expressing plasmids using Lipofectamine 2000 (Invitrogen, Carlsbad, Ca). Forty-eight hours later, transfectants were replated in triplicate and cultured for 10–15 days in complete medium containing G418. Surviving colonies were stained with crystal violet (0.5% w/v) after methanol fixation, with visible colonies (≥50 cells) counted.

### TUNEL assay

Cells cultured on coverslips were fixed with 4% paraformaldehyde, and permeabilized with 0.1% triton X-100. TUNEL (Terminal deoxynucleotidyl transferase dUTP nick end labeling) staining was performed using the *In Situ* Cell Death Detection Kit (Roche, Mannheim, Germany).

### Statistical analysis

Student’s t-tests were performed. All reported p-values were two-sided, and p < 0.05 was considered statistically significant.

## Additional Information

**How to cite this article**: Li, L. *et al*. Epigenetic inactivation of the CpG demethylase TET1 as a DNA methylation feedback loop in human cancers. *Sci. Rep.* **6**, 26591; doi: 10.1038/srep26591 (2016).

## Supplementary Material

Supplementary Information

## Figures and Tables

**Table 1:** Summary of *TET1* methylation in cell lines, tumor and normal tissues.

		Cell lines (% methylated)	Primary tumors (% methylated)
Carcinomas	Nasopharyngeal (NPC)	100% (5/5)	55% (31/56)
	Esophageal (ESCC)	50% (3 + 1w/8)	18% (7/38)
	Lung	80% (4/5)	13% (2/16)
	Gastric	92% (11 + 1w/16)	55% (30/55)
	Hepatocellular (HCC)	63% (5/8)	42% (5/12)
	Colorectal (CRC)	64% (6 + 1w/11)	27% (3/11)
	Breast	56% (4 + 1w/9)	36% (18/50)
	Cervical	75% (2 + 1w/4)	
	Renal	78% (6 + 1w/9)	28% (13/46)
	Prostate	33% (1/3)	22% (2/9)
Lymphomas	non-Hodgkin	85% (11/13)	eBL, 50% (3/6) DLBCL, 20%(2/10)
	Hodgkin	100% (8/8)	78% (5 + 2w/9)
	Nasal, NK/T-cell (NKTCL)	100% (4/4)	83% (10/12)
Screen tissue	Nose swab from NPC patients		50% (8/16)
Immortalized normal epithelial cell lines	NP460, NP69, Het-1A, NE1, NE3, NE083, HMEC, HMEpC, CCD841-CoN, HEK293, RHEK-1,	0 (0/11)	
Surgical margin tissues of tumors	breast tissues		20% (1/5)
Normal tissues	Normal nasopharynx (NPx)		0 (0/5)
	Normal breast tissues		0 (0/22)

W, weak methylation.

NPC, nasopharyngeal carcinoma; ESCC, esophageal squamous carcinoma; HCC, hepatocellular carcinoma; CRC, colorectal carcinoma; eBL, endemic Burkitt lymphoma; DLBCL, diffuse large B-cell lymphoma.
