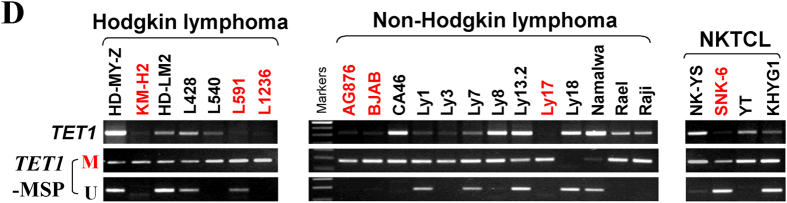# Corrigendum: Epigenetic inactivation of the CpG demethylase TET1 as a DNA methylation feedback loop in human cancers

**DOI:** 10.1038/srep34435

**Published:** 2016-10-06

**Authors:** Lili Li, Chen Li, Haitao Mao, Zhenfang Du, Wai Yee Chan, Paul Murray, Bing Luo, Anthony TC Chan, Tony SK Mok, Francis KL Chan, Richard F Ambinder, Qian Tao

Scientific Reports 6: Article number: 2659110.1038/srep26591; published online: 05 26 2016; updated: 10 06 2016.

This Article contains errors in Figure 2D where the Hodgkin lymphoma ‘TET1-MSP’ methylated and unmethylated MSP bands are incorrect. The correct Figure 2D appears below as Figure 1.

## Figures and Tables

**Figure 1:**