# Supplementary material for: Epigenetic inactivation of the CpG demethylase TET1 as a DNA methylation feedback loop in human cancers
Source: Sci Rep. 2016 May 26;6:26591. doi: 10.1038/srep26591 (PMC4880909; doi:10.1038/srep26591)
Supplement: Supplementary Information [file srep26591-s1.pdf]

Epigenetic inactivation of the CpG demethylase TET1 as a DNA methylation feedback loop in human cancers

Lili Li[1], Chen Li[1], Haitao Mao[1], Zhenfang Du[1], Wai Yee Chan[2], Paul Murray[3], Bing Luo[4], Anthony TC Chan[1], Tony SK Mok[1], Francis KL Chan[5], Richard F Ambinder[6], Qian Tao[1,6]

[1]Cancer Epigenetics Laboratory, Department of Clinical Oncology, State Key Laboratory of Oncology in South China, Sir YK Pao Center for Cancer, Li Ka Shing Institute of Health Sciences; [2]School of Biomedical Sciences, The Chinese University of Hong Kong, Hong Kong; [3]School of Cancer Sciences, University of Birmingham, Birmingham, UK; [4]Department of Medical Microbiology, Qingdao University Medical College, Shandong, China; [5]Institute of Digestive Disease and State Key Laboratory of Digestive Diseases, Department of Medicine and Therapeutics, The Chinese University of Hong Kong, Hong Kong; [6]Johns Hopkins Singapore and Sydney Kimmel Comprehensive Cancer Center, Johns Hopkins School of Medicine, Baltimore

Supplementary figure legends

Figure S1. *TET1* methylation in other tumor cell lines. (A) *TET2* and *TET3* are readily expressed in multiple carcinoma, non-Hodgkin (NHL) and nasal NK/T-cell (NKTCL) lymphoma cell lines except for two, detected by semi-quantitative RT-PCR with same cDNA samples as used in Figure 2. (B) Examination of *TET1* expression and methylation in hepatocellular, bladder, prostate cancer cell lines, and melanoma cell lines. Ca, carcinoma; NPC, nasopharyngeal carcinoma; ESCC, esophageal squamous cell carcinoma; CRC, colorectal cancer; HCC, hepatocellular carcinoma; CxCa, cervical cancer; RCC, renal cancer.

Figure S2. Detection of *TET1* methylation by MSP in primary tumors of esophageal (ESCC), lung, hepatocellular (HCC), prostate, endemic Burkitt lymphoma (eBL), and diffuse large B-cell lymphoma (DLBCL). Ca, carcinoma.

Figure S3. *TET1* expression was broadly reduced in multiple human cancers. Analysis of *TET1* mRNA expression using (A) the GENT and (B) the Oncomine databases. *TET1* was found to be significantly downregulated in different types of tumors, compared to normal controls. Ca, carcinoma; CRC, colon cancer; ccRCC, clear cell renal cell carcinoma; CxCa, cervical cancer; OvCa, ovarian cancer; CLL, chronic lymphocytic leukemia.

Figure S4. Genetic deletion analysis of *TET1* in multiple tumor cell lines. (A, B) *TET1* deletion was examined by multiplex differential genomic DNA-PCR using primers targeting two regions spanning exon 2 or exon 4, with *GAPDH* as an internal control. *TET1* expression and methylation status in each sample is shown in bottom panels. Red-colored cell lines are considered as with hemizygous deletions. +, expressed; d, downregulated or silenced; M, methylated; U, unmethylated. (C) Alterations of *TET1* DNA copy number in human cancers, analyzed using the Oncomine database. NPC, nasopharyngeal carcinoma; ESCC, esophageal squamous cell carcinoma; GsCa, gastric cancer; CRC, colorectal cancer; BrCa, breast cancer; CxCa, cervical cancer; RCC, renal cancer; NHL, non-Hodgkin lymphoma; HL, Hodgkin lymphoma; NKTCL, nasal NK/T-cell lymphoma; HNSCC, head and neck squamous cell carcinoma; HCC, hepatocellular carcinoma; Ca, carcinoma.

Supplementary Table S1. Sequences of primers used in the study

PCR	Primers	Sequence (5'-3')	Product size	Annealing Temp.(ºC)	Cycles
RT-PCR	*TET1*F	GTGCCAATGGTCAGTACCTC	329 bp	55	32
	*TET1*R	CTGTAGTCCATGGATTCTGA			
	*HOXA5*F	CTGGATGCGCAAGCTGCACA	251 bp	55	32
	*HOXA5*R	CCATGCTCATGCTTTTCAGC			
	*HOXA9*F	GTGTACCACCACCATCACCA	192 bp	55	32
	*HOXA9*R	AGCGCGCATGAAGCCAGTTG			
	*PCDH7*F	GTTCCGAGTACCTGAAGATC	208 bp	55	32
	*PCDH7*R	GTGTTGTCGTTGATGTCAAG			
	*TCF4*F	TAGGGACGGACAAAGAGCTG	168 bp	55	32
	*TCF4*R	TTGGATGTCCTCCATTCCCC			
	*MEIS1*F	GGAGCCAGAGAGGCCGATG	192 bp	55	32
	*MEIS1*R	ATGGCGTTGGTATGAGCTGTG			
	SLIT2F	TGCTGGCGATCCTGAACAAG	260 bp	55	32
	SLIT2R	AAGATCCTGGAATGCTCCTC			
	ZNF382F	CCTTACAGGGATCAGTGTCA	173 bp	55	32
	ZNF382R	CAACTTGCGGATCATATCAG			
	*GAPDH*55	ATCTCTGCCCCCTCTGCTGA	303 bp	55	23
	*GAPDH*33	GATGACCTTGCCCACAGCCT			
Multiplex genomic DNA-PCR	*TET1*F	GTGCCAATGGTCAGTACCTC	296 bp (exon 2)		
	*TET1Int2*R	GACACTAGAAGTGTCTCTGC			
	*TET1Int4*F	TGCGATTACAGGTGTGAGTC	281 bp (exon 4)		
	*TET1*R	CTGTAGTCCATGGATTCTGA			
	GAPDHInt7F	GCCTCACTCCTTTTGCAGAC	155 bp		
	*GAPDH*33	GATGACCTTGCCCACAGCCT			

MSP	*TET1*m4	GTCGGTAGGGTTTTTCGC	173 bp	60	40
	*TET1*m8	CCCAACTCACCGCTAACCG			
	*TET1*u4	GAGTTGGTAGGTGTTTTTTGT	175 bp	58	40
	*TET1*u8	CCCAACTCACCACTAACCA			
	*HOXA9*m3	ATTCGTTTTTGTTGGGCGTC			
	*HOXA9*m4	GCAACGAATACAACGTTAACG		60	40
	*HOXA9*u3	GGATTTGTTTTTGTTGGGTGTT			
	*HOXA9*u4	CCACAACAAATACAACATTAACA		58	40
	*SLIT2*m5	GATCGGTTTAGGTTGCGGC			
	*SLIT2*m7	AACAACTAAACATAACGCGCG		60	40
	*SLIT2*u5	GGATTGGTTTAGGTTGTGGT			
	*SLIT2*u7	AAAACAACTAAACATAACACACA		58	40
	*ZNF382*m3	GGCGATTAACGGGTCGTTTC			
	*ZNF382*m5	AAAATTTCCAAACCCGACTCG		60	40
	*ZNF382*u3	GTGGTGATTAATGGGTTGTTTT			
	*ZNF382*u5	CAAAATTTCCAAACCCAACTCA		58	40
BGS	*TET1*BGS6	TTGTTTTTTTATTGTGGATTTTTG	384 bp	60	40
	*TET1*BGS2	AACCCACCCCTAAAACAAC			
Cloning	*TET1*-CF	CGGAATTCGCCACCATGGATTACAAGGATGA CGACGATAAGTACCCATACGATGTTCCAGA	2266 bp	58	18
	*TET1-C*R	GCTCTAGATTAGTGGTGATGGTGATGATGGA CCCAATGGTTATAGGGC			

Suppl fig1

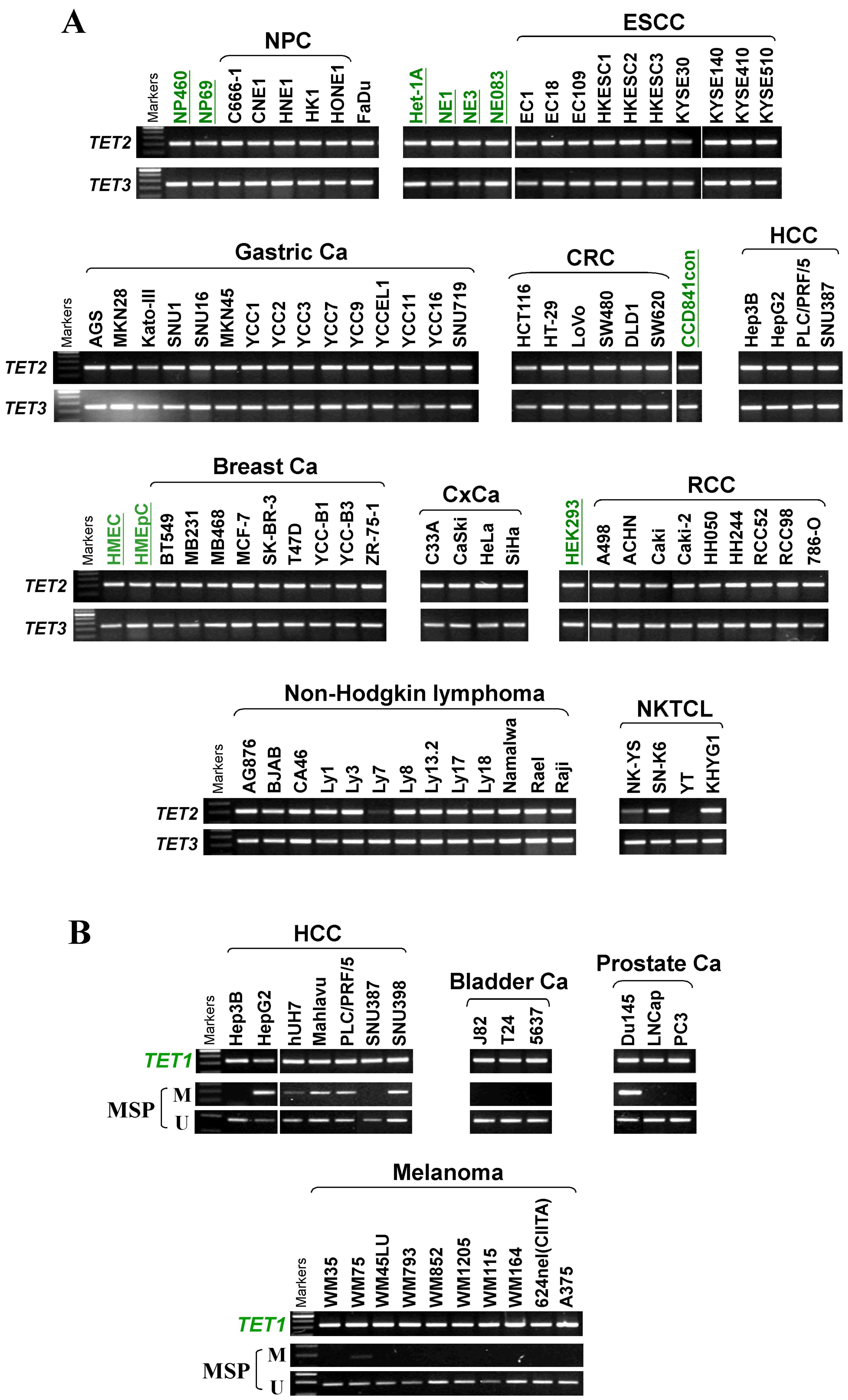

A
NPC
ESCC
Markers
NP460
NP69
C666-1
CNE1
HNE1
HK1
HONE1
FaDu
Het-1A
NE1
NE3
NE083
EC1
EC18
EC109
HKESC1
HKESC2
HKESC3
KYSE30
KYSE140
KYSE410
KYSE510
TET2
TET3
Gastric Ca
AGS
MKN28
Kato-III
SNU1
SNU16
MKN45
YCC1
YCC2
YCC3
YCC7
YCC9
YCCEL1
YCC11
YCC16
SNU719
CRC
HCT116
HT-29
LoVo
SW480
DLD1
SW620
CCD841con
HCC
Hep3B
HepG2
PLC/PRF/5
SNU387
Breast Ca
HMEC
HMEpC
BT549
MB231
MB468
MCF-7
SK-BR-3
T47D
YCC-B1
YCC-B3
ZR-75-1
CxCa
C33A
CaSki
HeLa
SiHa
RCC
HEK293
A498
ACHN
Caki
Caki-2
HH050
HH244
RCC52
RCC98
786-O
Non-Hodgkin lymphoma
AG876
BJAB
CA46
Ly1
Ly3
Ly7
Ly8
Ly13.2
Ly17
Ly18
Namalwa
Rael
Raji
NKTCL
NK-YS
SN-K6
YT
KHYG1
B
HCC
Hep3B
HepG2
hUH7
Mahlavu
PLC/PRF/5
SNU387
SNU398
Bladder Ca
J82
T24
5637
Prostate Ca
Du145
LNCap
PC3
TET1
MSP
M
U
Melanoma
WM35
WM75
WM45LU
WM793
WM852
WM1205
WM115
WM164
624nel(CIITA)
A375

Suppl fig2

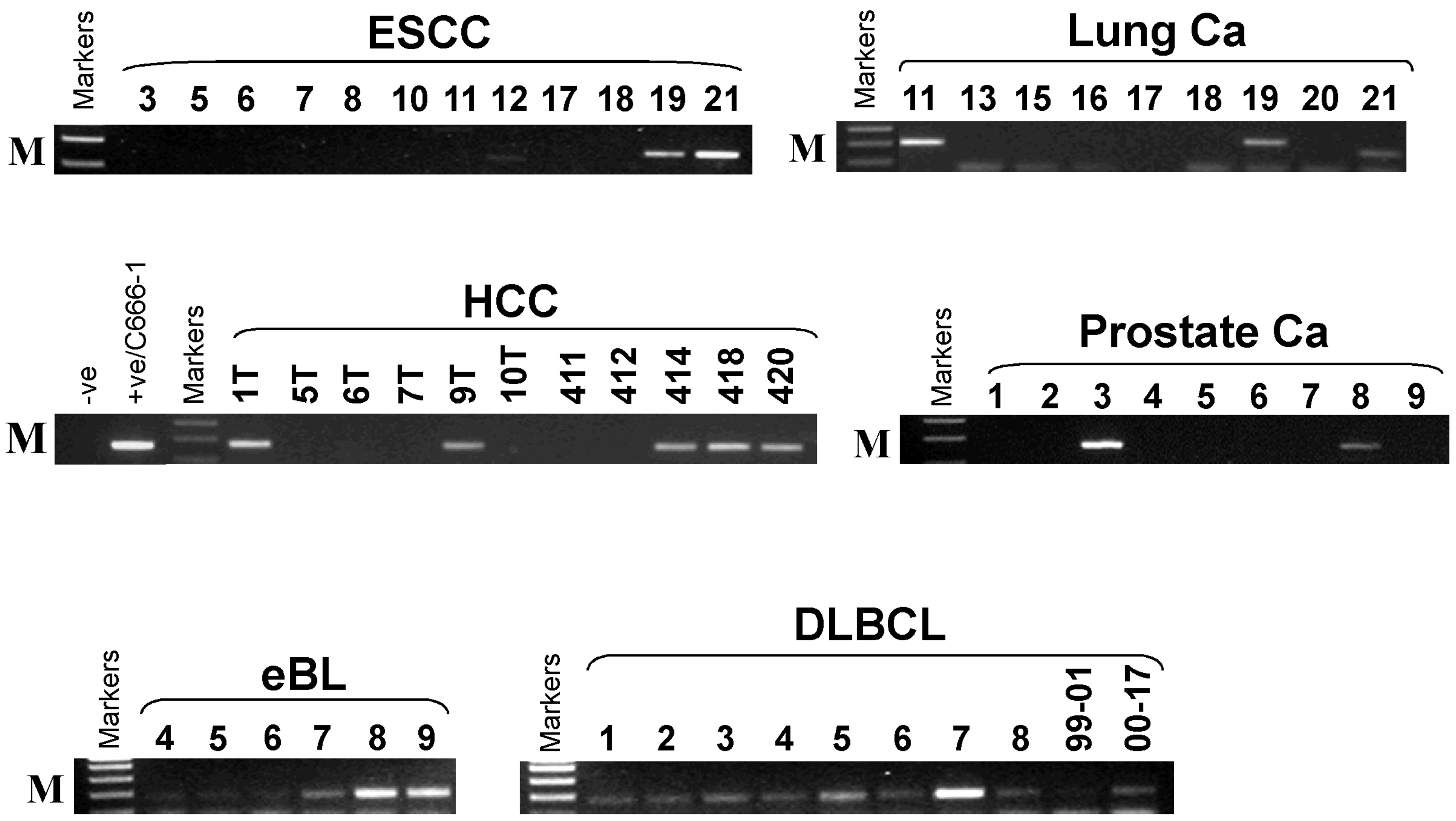
ESCC
Markers
3 5 6 7 8 10 11 12 17 18 19 21
M
Lung Ca
Markers
11 13 15 16 17 18 19 20 21
M
HCC
-ve
+ve/C666-1
Markers
1T 5T 6T 7T 9T 10T 411 412 414 418 420
M
Prostate Ca
Markers
1 2 3 4 5 6 7 8 9
M
eBL
Markers
4 5 6 7 8 9
M
DLBCL
Markers
1 2 3 4 5 6 7 8 99-01 00-17

Suppl fig3

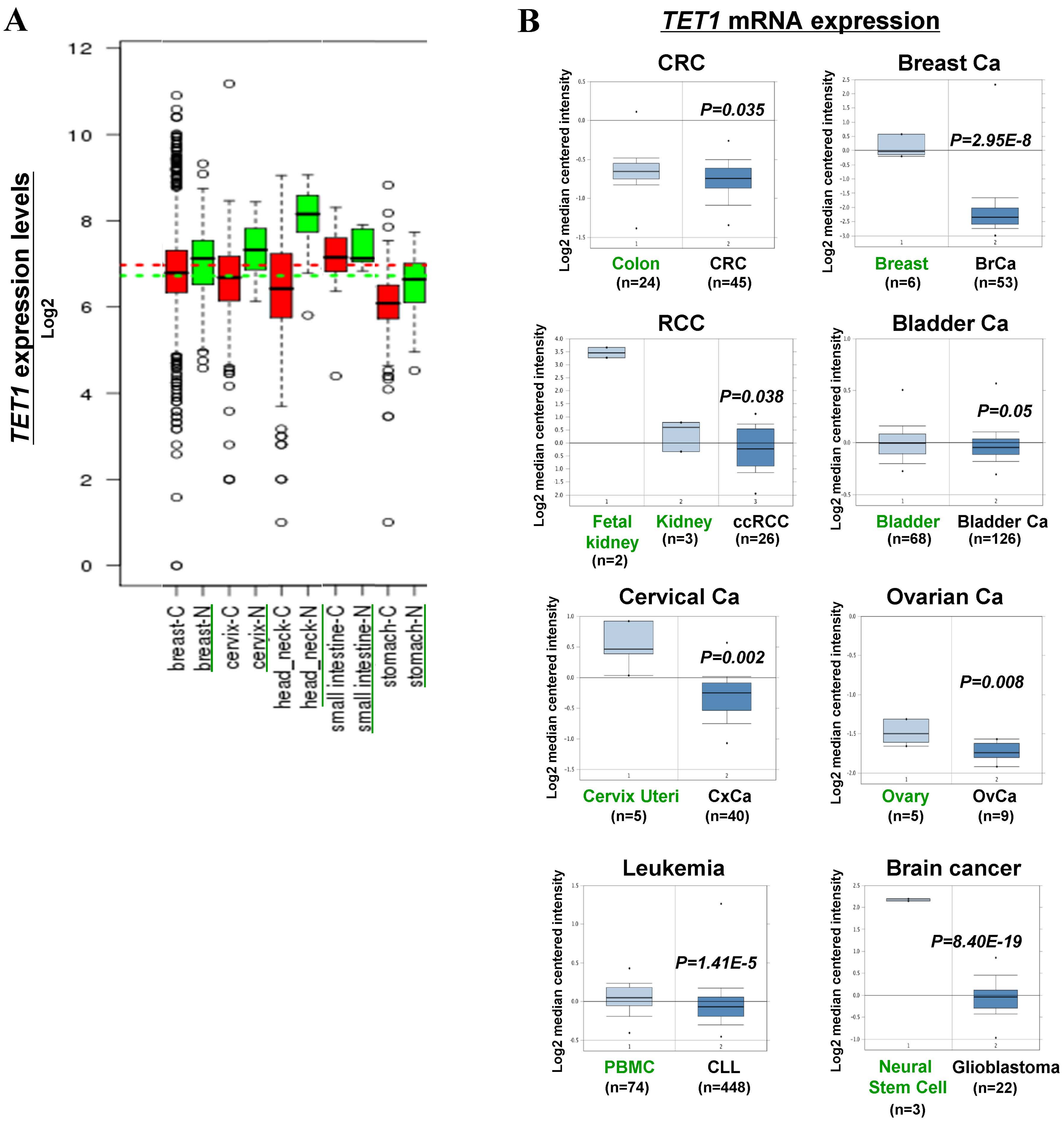
A
TET1 expression levels
Log2
breast-C
breast-N
cervix-C
cervix-N
head_neck-C
head_neck-N
small intestine-C
small intestine-N
stomach-C
stomach-N
B
TET1 mRNA expression
CRC
Colon (n=24)
CRC (n=45)
P=0.035
Breast Ca
Breast (n=6)
BrCa (n=53)
P=2.95E-8
RCC
Fetal kidney (n=2)
Kidney (n=3)
ccRCC (n=26)
P=0.038
Bladder Ca
Bladder (n=68)
Bladder Ca (n=126)
P=0.05
Cervical Ca
Cervix Uteri (n=5)
CxCa (n=40)
P=0.002
Ovarian Ca
Ovary (n=5)
OvCa (n=9)
P=0.008
Leukemia
PBMC (n=74)
CLL (n=448)
P=1.41E-5
Brain cancer
Neural Stem Cell (n=3)
Glioblastoma (n=22)
P=8.40E-19
Log2 median centered intensity

Suppl fig4

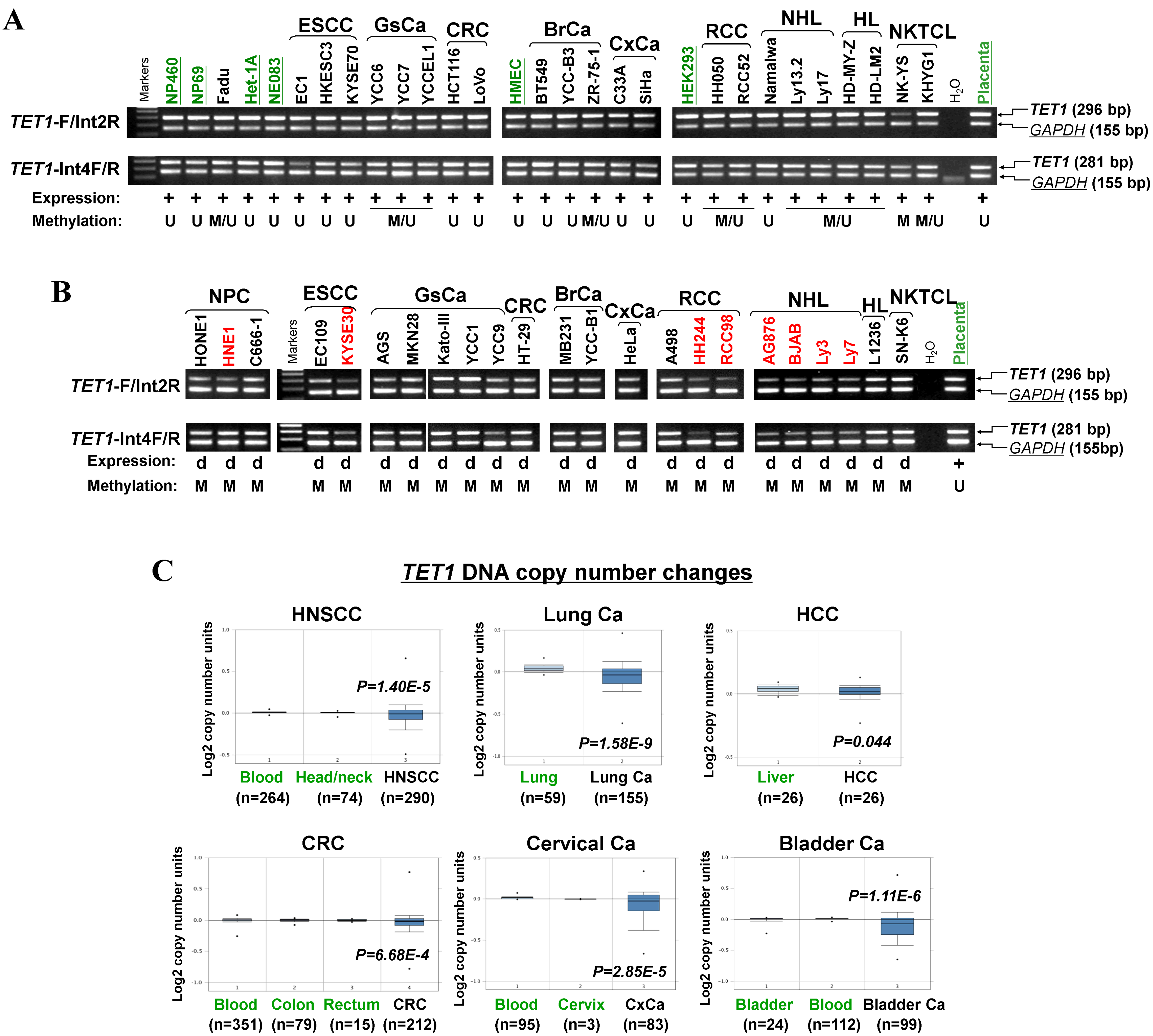
A
ESCC
GsCa
CRC
BrCa
CxCa
RCC
NHL
HL
NKTCL
Markers
NP460
NP69
Fadu
Het-1A
NE083
EC1
HKESC3
KYSE70
YCC6
YCC7
YCCEL1
HCT116
LoVo
HMEC
BT549
YCC-B3
ZR-75-1
C33A
SiHa
HEK293
HH050
RCC52
Namalwa
Ly13.2
Ly17
HD-MY-Z
HD-LM2
NK-YS
KHYG1
H2O
Placenta
TET1-F/Int2R
TET1-Int4F/R
TET1 (296 bp)
GAPDH (155 bp)
TET1 (281 bp)
GAPDH (155 bp)
Expression:
Methylation:
B
NPC
ESCC
GsCa
CRC
BrCa
CxCa
RCC
NHL
HL
NKTCL
HONE1
HNE1
C666-1
Markers
EC109
KYSE30
AGS
MKN28
Kato-III
YCC1
YCC9
HT-29
MB231
YCC-B1
HeLa
A498
HH244
RCC98
AG876
BJAB
Ly3
Ly7
L1236
SN-K6
H2O
Placenta
TET1-F/Int2R
TET1-Int4F/R
TET1 (296 bp)
GAPDH (155 bp)
TET1 (281 bp)
GAPDH (155bp)
Expression:
Methylation:
C
TET1 DNA copy number changes
HNSCC
Lung Ca
HCC
Log2 copy number units
P=1.40E-5
P=1.58E-9
P=0.044
Blood (n=264)
Head/neck (n=74)
HNSCC (n=290)
Lung (n=59)
Lung Ca (n=155)
Liver (n=26)
HCC (n=26)
CRC
Cervical Ca
Bladder Ca
P=6.68E-4
P=2.85E-5
P=1.11E-6
Blood (n=351)
Colon (n=79)
Rectum (n=15)
CRC (n=212)
Blood (n=95)
Cervix (n=3)
CxCa (n=83)
Bladder (n=24)
Blood (n=112)
Bladder Ca (n=99)
